# DeepLabv3 + method for detecting and segmenting apical lesions on panoramic radiography

**DOI:** 10.1007/s00784-025-06156-0

**Published:** 2025-01-31

**Authors:** Fatmanur Ketenci Çay, Çağrı Yeşil, Oktay Çay, Büşra Gül Yılmaz, Fatma Hasene Özçini, Dilhan İlgüy

**Affiliations:** 1 Department of Dentomaxillofacial Radiology, Faculty of Dentistry, Yeditepe University, Istanbul, Turkey; 2 SADC Department, Huawei Türkiye R&D Center, Istanbul, Turkey; 3 Şakir Gürkan Family Health Center, Istanbul, Turkey; 4 Department of Dentomaxillofacial Radiology, Faculty of Dentistry, Sağlık Bilimleri University, Istanbul, Turkey; 5 Faculty of Dentistry, Yeditepe University, Caddebostan, Bağdat St. Nu:238, Kadıköy, İstanbul, 34728 Turkey; 6 Huawei Türkiye, R&D Center, Ümraniye, Istanbul, Turkey; 7 Fındıklı, Gazi Mustafa Kemal St. Nu:23, Maltepe, İstanbul, 34854 Turkey; 8 Mekteb-i Tıbbiye-i Şahane (Hamidiye) Külliyesi Selimiye, Tıbbiye St. Nu:38, Üsküdar, İstanbul, 34668 Turkey; 9 Mehmet Akif, Tacirler Eğitim Vakfı Sultanbeyli Devlet Hst., Sultanbeyli, İstanbul, 34920 Turkey

**Keywords:** Apical lesions, Semantic segmentation, Deep learning, Artificial intelligence

## Abstract

**Objective:**

This study aimed to apply the DeepLabv3 + model and compare it with the U-Net model in terms of detecting and segmenting apical lesions on panoramic radiography.

**Methods:**

260 panoramic images that contain apical lesions in different regions were collected and randomly divided into training and test datasets. All images were manually annotated for apical lesions using Computer Vision Annotation Tool software by two independent dental radiologists and a master reviewer. The DeepLabv3 + model, one of the state-of-the-art deep semantic segmentation models, was utilized using Python programming language and the TensorFlow library and applied to the prepared datasets. The model was compared with the U-Net model applied to apical lesions and other medical image segmentation problems in the literature.

**Results:**

The DeepLabv3 + and U-Net models were applied to the same datasets with the same hyper-parameters. The AUC and recall results of the DeepLabv3 + were 29.96% and 61.06% better than the U-Net model. However, the U-Net model gets 69.17% and 25.55% better precision and F1-score results than the DeepLabv3 + model. The difference in the IoU results of the models was not statistically significant.

**Conclusions:**

This paper comprehensively evaluated the DeepLabv3 + model and compared it with the U-Net model. Our experimental findings indicated that DeepLabv3 + outperforms the U-Net model by a substantial margin for both AUC and recall metrics. According to those results, for detecting apical lesions, we encourage researchers to use and improve the DeepLabv3 + model.

**Clinical relevance:**

The DeepLabv3 + model has the poten tial to improve clinical diagnosis and treatment planning and save time in the clinic.

## Introduction

Apical periodontitis is characterized by an inflammation that occurs in the apical region of the root of a tooth, primarily due to bacterial infection within the pulp of the tooth, leading to a subsequent inflammatory response in the surrounding bone tissue [1]. On radiographs, apical periodontitis can be recognized by the appearance of periapical radiolucency, which may manifest as either a widened periodontal ligament or a lesion [2, 3]. These radiolucencies, called apical lesions (AL), can be identified by focused radiographic examinations. Panoramic and periapical radiographs are the most widely used techniques to diagnose and treat apical lesions [4].

However, apical lesions can easily be overlooked in dental radiographs in clinics because of the complex anatomy of the jaws and teeth, especially due to superimpositions of the anatomical structures onto the apical lesions. Moreover, dentists often review numerous radiographs daily, which can lead to visual fatigue and reduced concentration over time. This fatigue can decrease the accuracy of lesion segmentation, especially toward the end of the day or after reviewing many images in succession. Also, due to the daily workload of the clinics, radiographs may not be evaluated comprehensively, and therefore an incorrect or incomplete diagnosis may be made. Depending on this, various complications such as abscess formation, cystification, a larger alveolar bone defect, and even tooth loss may occur. Such cases require AI-based computer-aided diagnosis tools that can assist dentists in interpreting medical images.

AI-based segmentation of apical lesions can help differentiate radiolucencies caused by anatomical structures from true apical lesions on panoramic radiographs, thereby reducing the risk of misdiagnosis [3]. Segmentation enables the precise measurement of lesion size, which is crucial for diagnosing the extent of apical periodontitis, planning appropriate treatment strategies, and monitoring the disease’s progression or resolution over time. Moreover, accurate segmentation can improve the quantification of treatment outcomes, providing objective data to support clinical decisions.

AI applications and segmentation have expanded rapidly in various fields, including medical management and medical imaging [5]. For instance, AI models such as OCR-Net, DeepLabv3+ [6], Feature Pyramid Network [7], YOLOv3, Faster R-CNN, RetinaNet, ResNext, DenseNet, and ShuffleNet [8] have been employed for the segmentation and classification of dental caries. In the context of periodontal bone loss detection, AI models such as DeepLabv3+ [9, 10], DeNTNet, R-CNN, and Faster R-CNN [11] have been utilized. Similarly, tooth segmentation has been addressed through models such as U-Net, Faster R-CNN [12], U-Net3+ [13], U-Net++, DeepLabV3+, MultiResU-Net, ResU-Net++, PraNet, Poly-PVT, TransResU-Net [14], and SegNet [15]. However, current research on the segmentation of apical lesions has primarily focused on the use of the U-Net model [2, 3, 16–19]. Although only one study [16] investigated Lightweight CNN for apical lesion segmentation, this study also used the U-Net model to enhance precision in lesion segmentation.

DeepLabv3+ [20] is another pixel-based image segmentation model widely used in medical imaging, capable of segmenting various medical images, including radiographs, MRI, and CT scans, as well as identifying tumors, organs, and jawbone lesions [21, 22]. DeepLabv3 + and U-Net are powerful tools in segmentation tasks. DeepLabv3 + is optimized for more generalized tasks, leveraging atrous convolution to adjust the field of view, which makes it effective across diverse image resolutions. On the other hand, U-Net, specifically designed for medical image segmentation, often delivers better performance in medical scenarios requiring high precision for small-scale segmentations [23].

However, DeepLabv3 + has not been used before to detect apical lesions, and its performance has not been compared with U-Net, yet. Determining which segmentation method is more successful and utilizing new models such as DeepLabv3 + is important for enhancing the reliability and success of artificial intelligence. Thus, apical lesions will be diagnosed promptly and accurately and treated at an early stage. In this way, the patient will be protected from complications that may arise due to delays in treatment, and the treatment procedure will be carried out in a more comfortable process for the patient. Herewith, the time spent in the clinics will be saved, and the treatment cost will be reduced.

This study focuses on the success of the DeepLabV3 + model in apical lesion segmentation and compares it with U-Net. This study aims to investigate whether the success of apical lesion segmentation can be improved with DeepLabV3+.

## Materials and methods

This study is reported following the Strengthening the Reporting of Observational Studies in Epidemiology (STROBE) guideline. The study design was authorized by the Non-Interventional Clinical Research Ethics Committee of Yeditepe University (decision date and number: 08.12.2023/ E.83321821-805.02.03-326). The study was conducted following the regulations of the Declaration of Helsinki.

The panoramic radiographs used in this study were randomly selected from the archives of the Faculty of Dentistry at Yeditepe University. The Morita Veraviewepocs (Kyoto, Japan) panoramic imaging system was used to obtain panoramic radiographs with the following parameters: 76 kVp, 7 mA, and 14s. Only the radiographs of patients with mixed dentition were excluded. Additionally, images with issues such as blurriness, distortion, or technician-related errors were excluded. After the exclusion of the radiographs, the records were checked to identify the gender and age of the participants. It was noted that 133 (51.15%) of the radiographs belonged to male patients and 127 (48.85%) belonged to females. Also, the ages of the participants whose panoramic radiographs were included ranged from 17 to 90 years, with a mean age of 48.9 years.

**Fig. 1:**
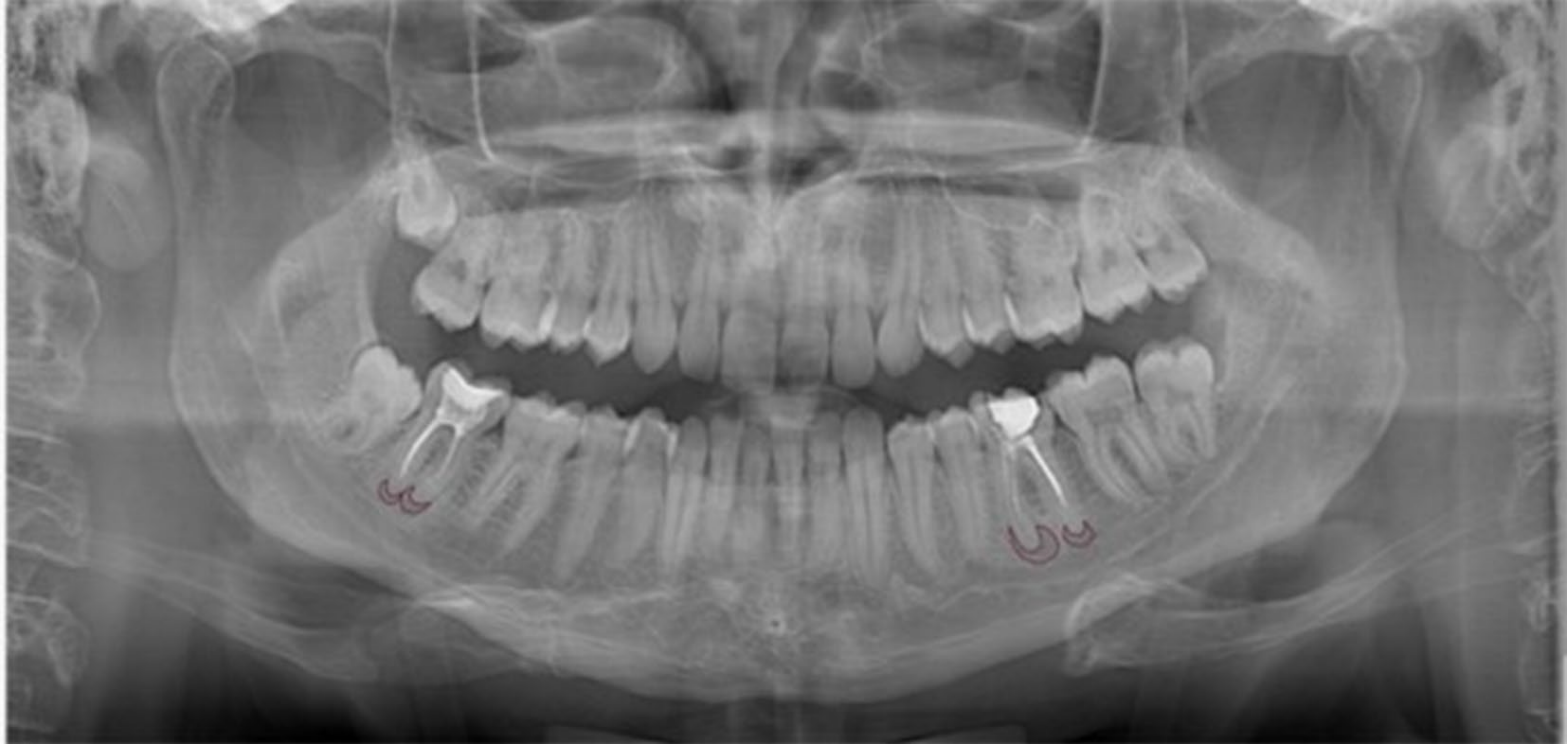
Annotation of the apical lesions

The selected images were cropped to remove the patients’ names, thereby ensuring patient privacy. A total of 260 anonymized panoramic radiographs were manually annotated using the Computer Vision Annotation Tool (CVAT) software by two independent dental radiologists (B.G.Y. and F.H.Ö. with five years of experience) and a master reviewer (F.K.Ç. with ten years of experience). The apical lesions were labeled using polygon labeling tool. An example of an annotated image is given in Fig. 1.

All images with different resolutions were collected, resized to the same size (512 × 512) to be able to be used in the models, and converted to PNG format. No augmentation strategy was applied to the images. The dataset was split into train and test by using the Kfold (k = 5) method of the Sklearn library. Therefore, 208 of 260 images were used in training, and 52 instances were used in the test set. In detail, the dataset was split into 5 different subsets randomly. In each trial, one set was used as the test set and others were used in the training.

**Fig. 2:**
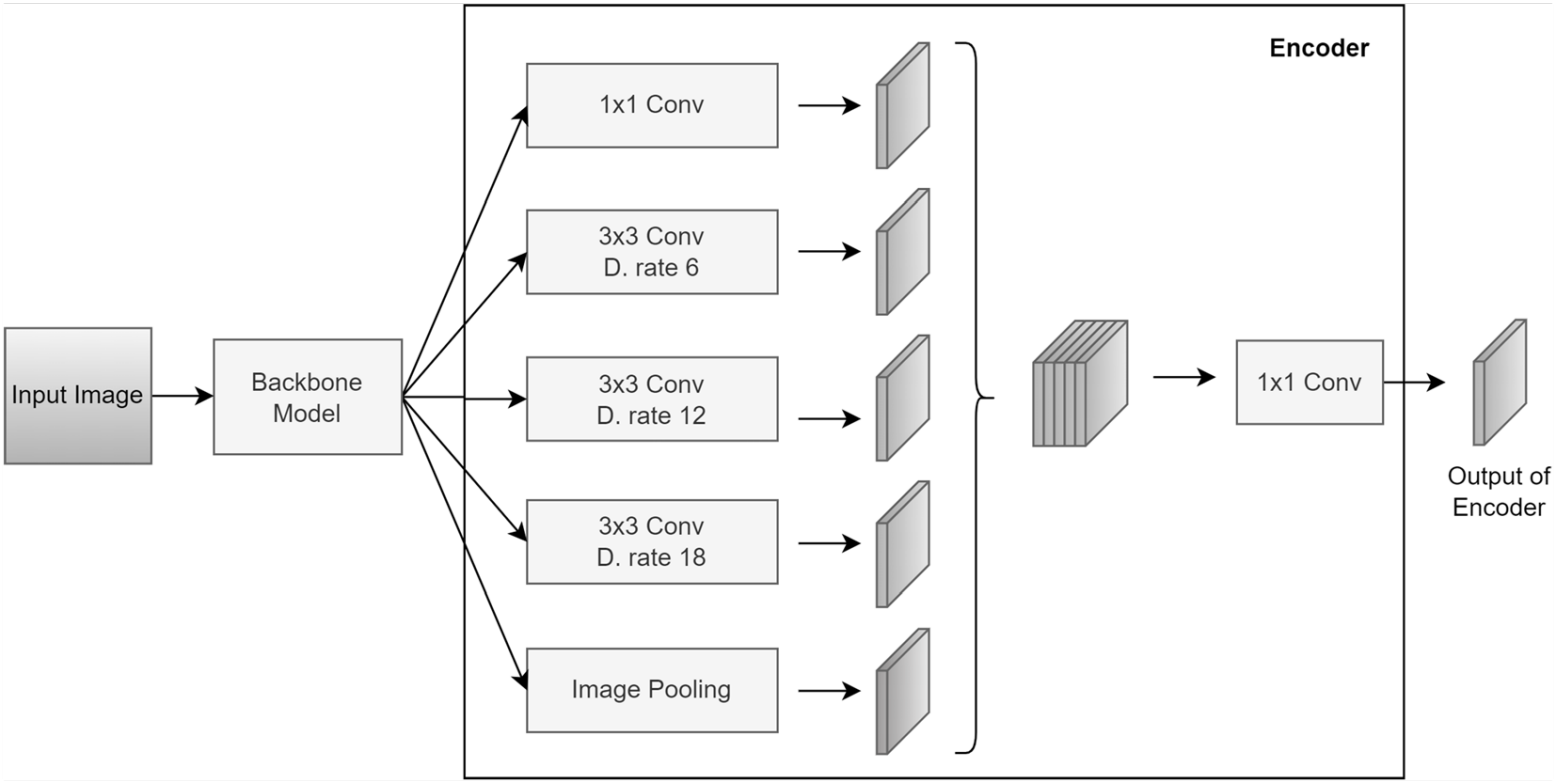
Encoder architecture of DeepLabv3+

This study employed a DeepLabv3 + network from the official Keras GitHub repository to segment apical lesions in panoramic images. The model features an encoder-decoder architecture utilizing atrous convolution. The encoder maps input radiographic images to a latent space through parallel convolution blocks with varying sizes, dilation rates, batch normalization, ReLU activation, and added dropout layers to prevent overfitting. Outputs are combined via pooling, concatenation, and 1 × 1 convolution (Fig. 2). As the original DeepLabv3 + paper suggested [20], transfer learning with ResNet50 [24], trained on ImageNet, was used to extract low-level features from specific layers for both encoding (conv4_block6_2_relu) and decoding (conv2_block3_2_relu).

**Fig. 3:**
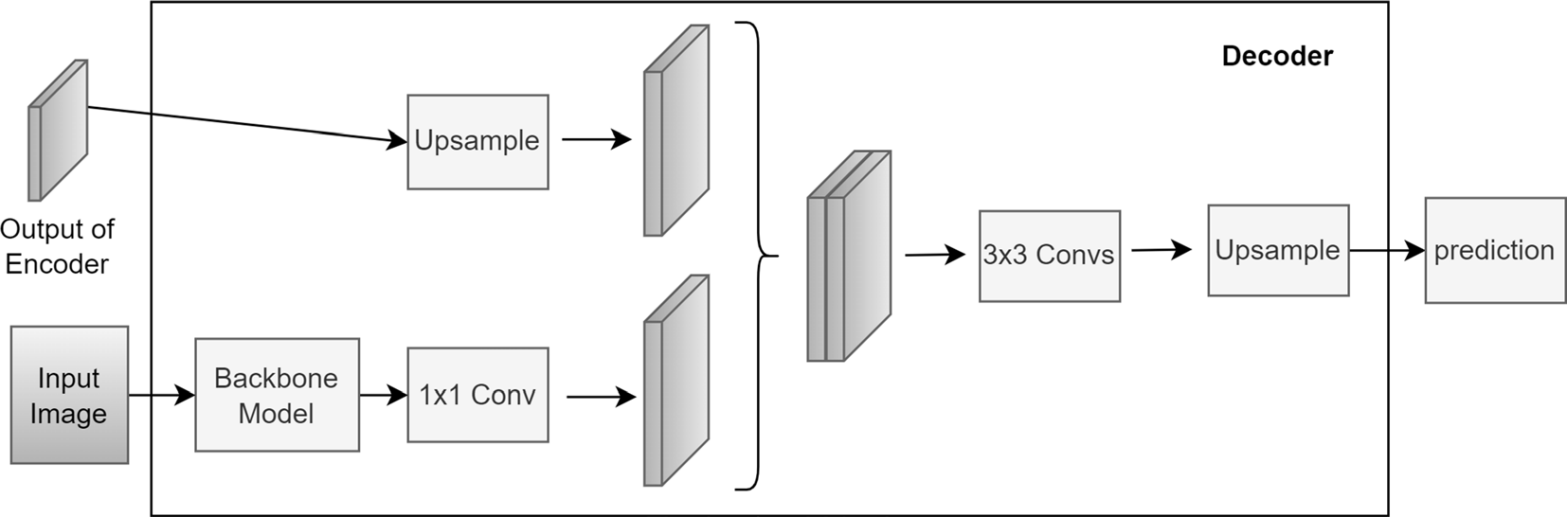
Decoder architecture of DeepLabv3+

The decoder reconstructs the segmentation mask by upsampling, convolving, and combining encoder outputs. Two final convolution layers and an up-convolution operation generate the prediction mask (Fig. 3). This process reverses the encoder’s contraction, translating latent information into the final segmentation mask.

**Fig. 4:**
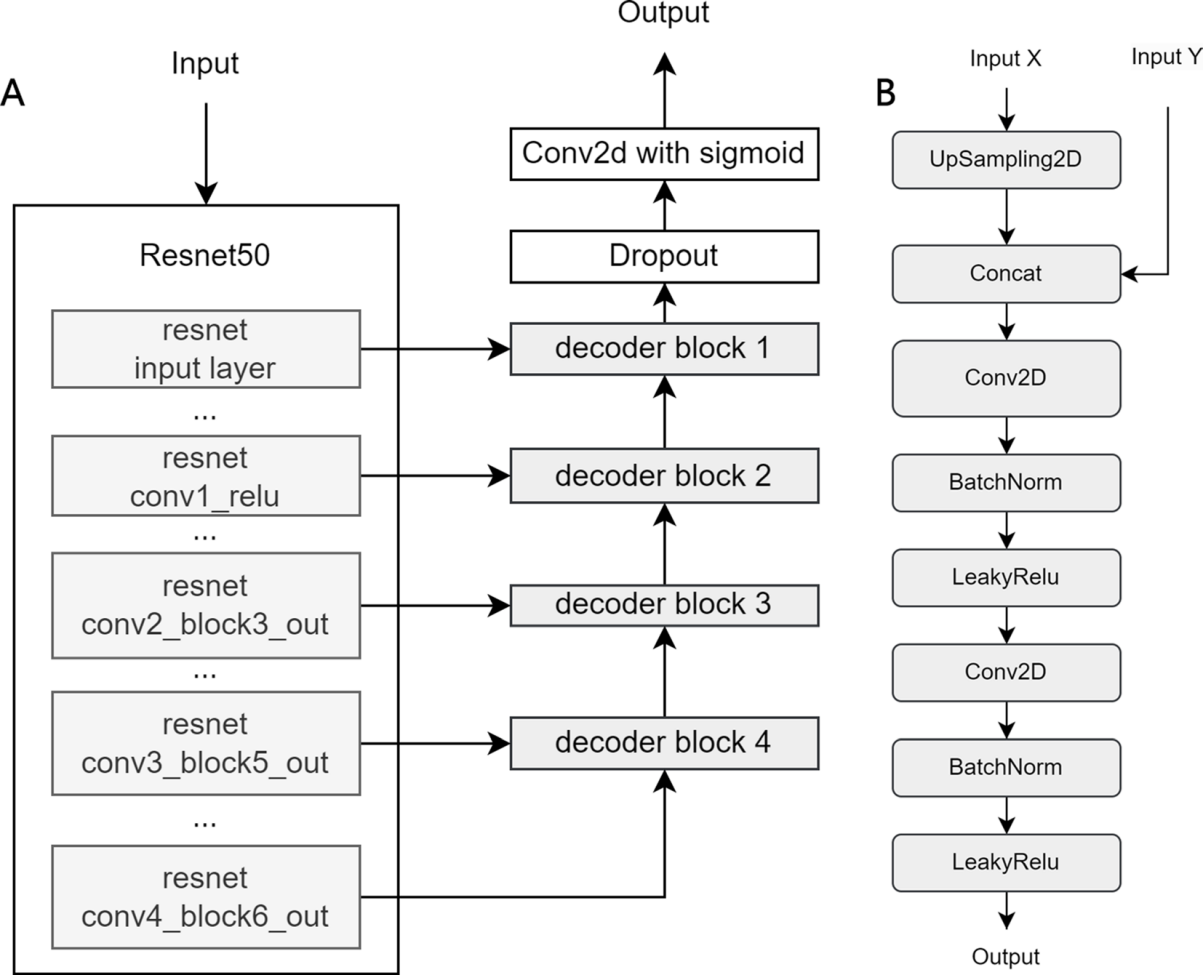
Architecture of U-Net. (**A**) U-Net Architecture, (**B**) Decoder Block

On the other hand, U-Net model used in comparison with DeepLabv3 + in this study, features an encoding path for context capture and a symmetric decoding path for precise localization. The encoder uses convolutional and pooling layers to extract features, while the decoder upsamples and combines feature maps to restore spatial details. Transfer learning with ResNet50 was applied to the encoder for a fair comparison. The detailed architecture is shown in Fig. 4.

The performance of the developed model was evaluated using the test set. Precision, recall, F1-score, and IoU metrics were utilized for evaluation, and they are demonstrated in the following Eqs. (1–4).

1 $$\:Precision=\frac{TP}{TP+FP\:}$$

2 $$\:Recall=\frac{TP}{TP+FN\:}$$

3 $$\:F1\_Score=2*\frac{Precision*Recall}{Precision+\:Recall\:}$$

4 $$\:IOU=\frac{TP}{TP+FN+FP\:}$$

TP, FP, and FN are the numbers of true positives, false positives, and false negatives, respectively. In addition to these parameters, the ROC-AUC, the area under the ROC curve, was used to evaluate the model.

## Results

The performance of the DeepLabv3 + model for apical lesion segmentation was evaluated using the performance metrics mentioned above. Additionally, its performance was compared with that of the U-Net model, which is commonly used for the segmentation of apical lesions. For a fair comparison, the same hyper-parameters were used for both models, as shown in Table 1.

The models were optimized using TensorFlow (v2.12.0) with CUDA (v12.2) in Python open-source programming language (v 3.9.16). The training and testing were processed with a computer having NVIDIA GeForce RTX 3060 with 12 GB VRAM, 13th Gen Intel(R) Core (TM) i7-13700 K 3.40 GHz, and 32 GB of DDR4 RAM.

**Table 1:** Hyper-parameters

Metric	Value
Batch_size	16
Learning_rate	0.001
Epoch	100
Dropout_rate	0.3
Loss Function	BCE
Optimizer	Adam

The performance results of the DeepLabv3 + and U-Net models were obtained by using 5-fold cross-validation. Table 2 shows the average of the folds with the corresponding standard deviation of DeepLabv3 + over U-Net. The better result for each metric is marked in bold. As seen in Table 2, the DeepLabv3 + model has 29.96% better AUC and 61.06% better recall results than the U-Net model. However, the U-Net model gets 3.61%, -69.17%, and 25.55% better IoU, precision, and F1-score results than the DeepLabv3 + model, respectively.

**Table 2:** Comparison of DeepLabv3 + and U-Net models

Metric	DeepLabv3+	U-Net	Rel. Imp.
AUC	**0.879 ± 0.055**	0.676 ± 0.051	29.96%
IoU	0.546 ± 0.011	**0.566 ± 0.032**	-3.61%
Precision	0.171 ± 0.074	**0.554 ± 0.122**	-69.17%
Recall	**0.248 ± 0.108**	0.154 ± 0.079	61.06%
F1-score	0.172 ± 0.036	**0.231 ± 0.096**	-25.55%

**Fig. 5:**
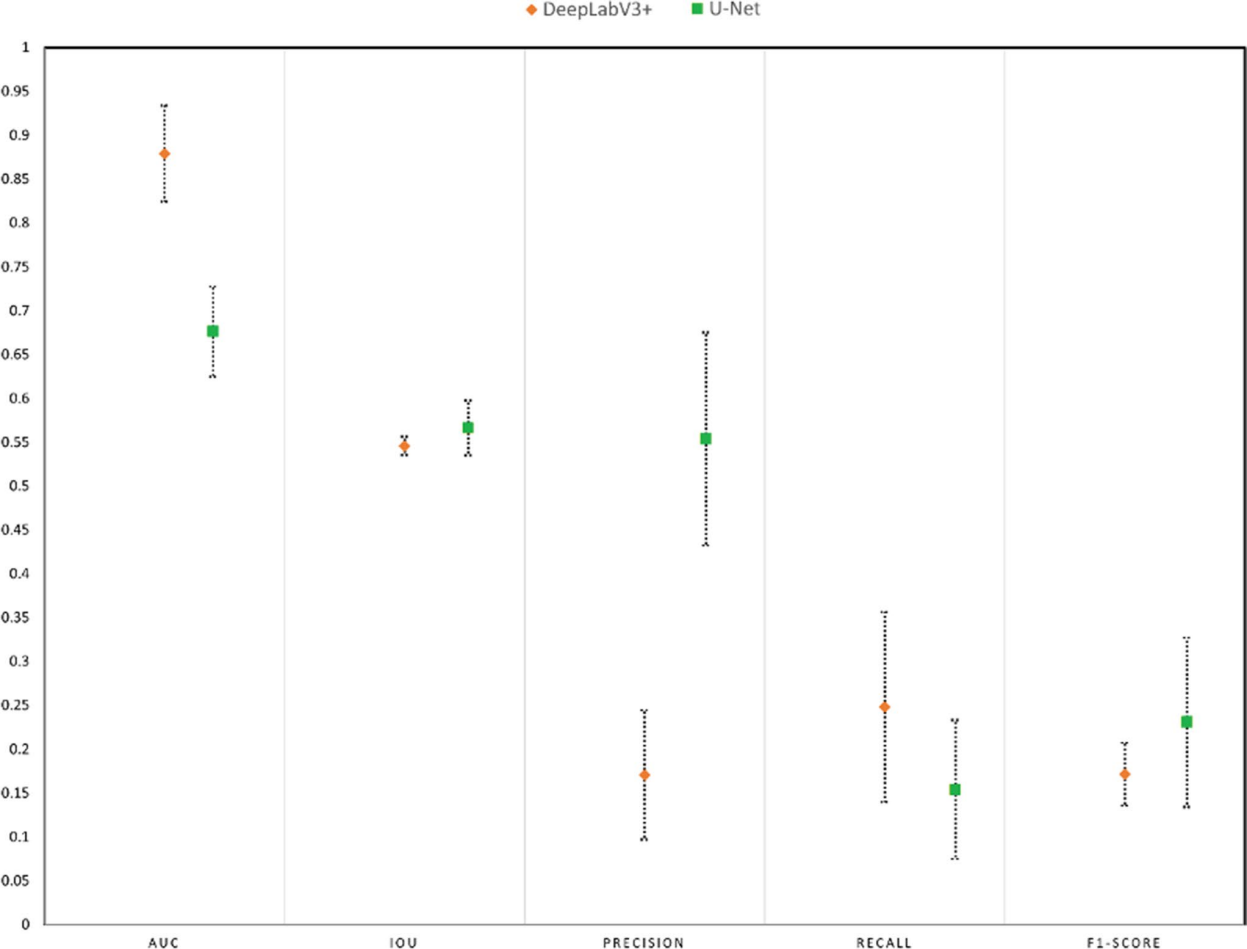
Comparison of results with error bars

The results and corresponding standard deviations are shown as error bars in Fig. 5 to interpret the effect of the standard deviation. Figure 5 shows that the minimum AUC value of the DeepLabv3 + model is larger than the maximum AUC value of the U-Net model. On the contrary, the minimum precision value of the U-Net model is larger than the maximum precision value of the DeepLabv3 + model. However, according to the error bar of the IoU scores, the difference between the DeepLabv3 + and U-Net models is not significant in the IoU metric. When the F1-score of the models is compared according to Fig. 5, it is seen that the minimum and maximum bars of the U-Net and DeepLabv3 + models intersect each other, which shows that the DeepLabv3 + model has the potential to get better F1-score results than the U-Net model.

We also carried out the t-test analysis by setting the significance threshold at 0.05 to compare the results of the U-Net and DeepLabv3 + models. According to the results of the t-test, the p-values were approximately 0.00065 for AUC, 0.26631 for IoU, 0.00125 for precision, 0.20123 for recall, and 0.30324 for F1-score. All p-values of AUC and precision were less than 0.05, meaning that performance differences between models were statistically significant for those metrics. But IoU, recall, and F1-score, p-values were larger than 0.05, which means that performance differences between DeepLabv3 + and U-Net were not statistically significant for these metrics.

**Fig. 6:**
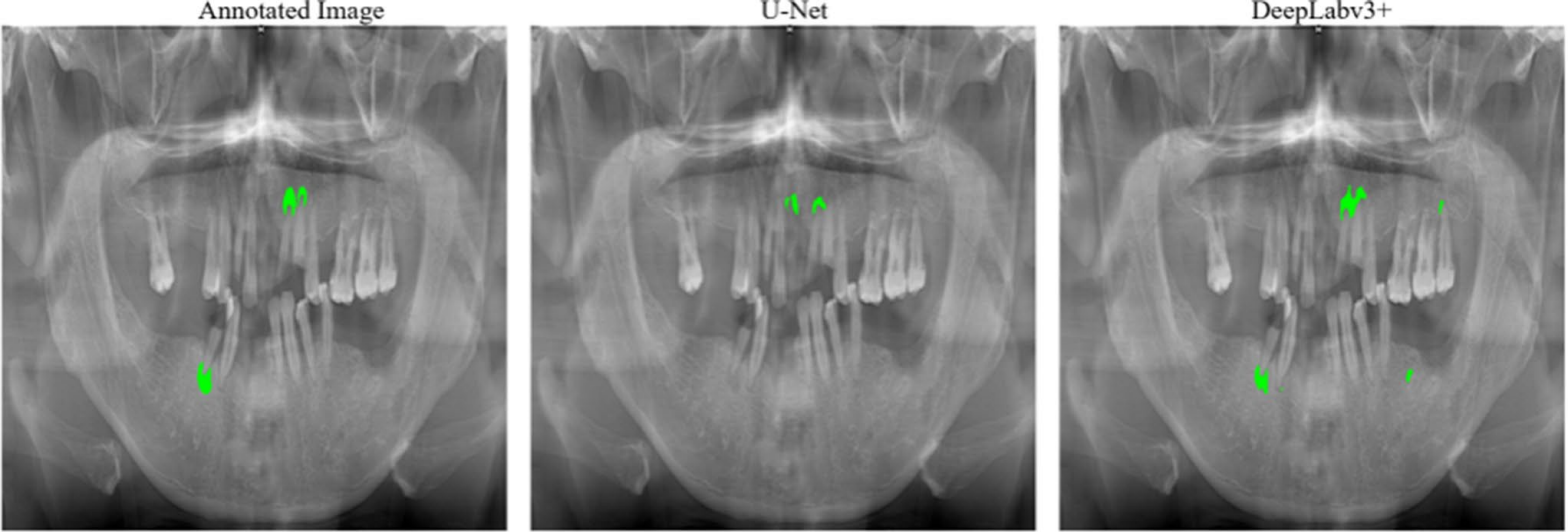
Comparison of U-Net and DeepLabv3 + outputs for an image in the test set where DeepLabv3 + has better prediction

**Fig. 7:**
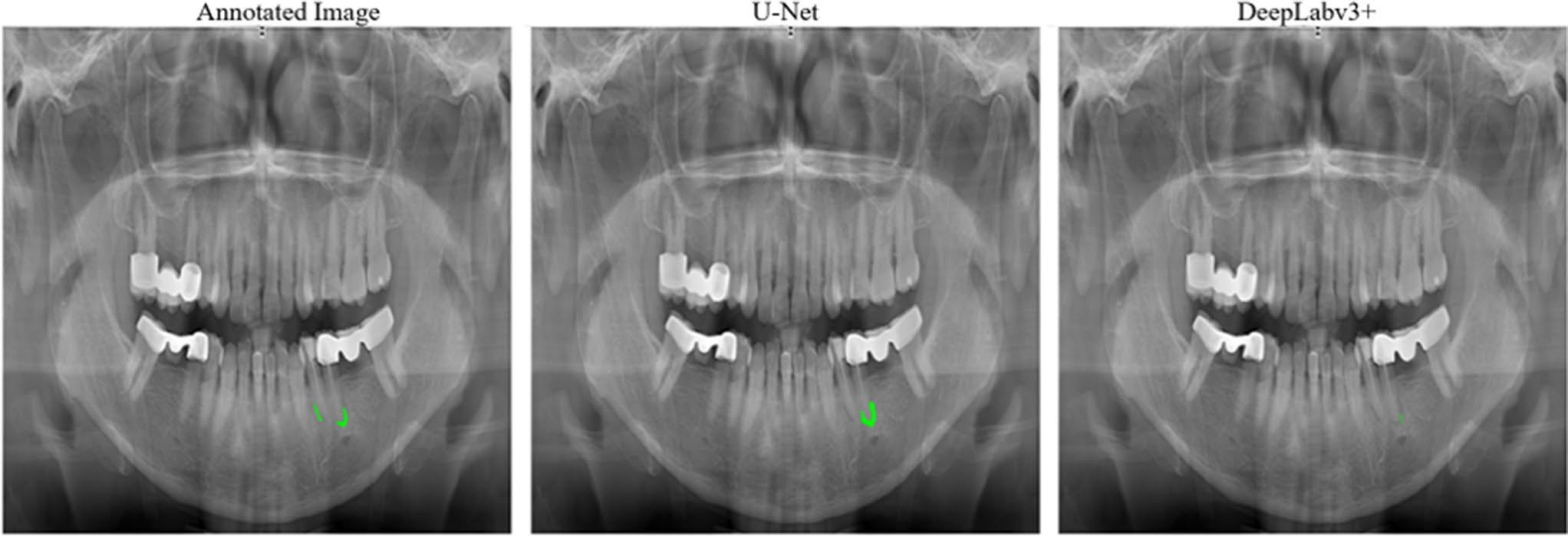
Comparison of U-Net and DeepLabv3 + outputs for an image in the test set where U-Net has better prediction

When we examined the outputs of both models, we observed that while some annotated apical lesions were better segmented by U-Net, others were better segmented by DeepLabv3+. The example outputs of both models from the test set are shown in Figs. 6 and 7.

## Discussion

In this study, we focused on the segmentation of apical lesions using the DeepLabv3 + artificial intelligence model and compared it with the U-Net model. The DeepLabv3 + model outperformed the U-Net model in terms of AUC and recall. However, the U-Net model achieved better results in precision, and F1-score compared to the DeepLabv3 + model.

The recall metric also known as true positive rate gives insight into a model’s prediction performance of true positives. From the perspective of apical lesion segmentation, a high recall means that the model can capture the region with a lesion more accurately. This increases the chance of early diagnosis which is crucial in the health domain. Moreover, high precision may reduce a model’s false positive rate, preventing false diagnosis. Based on our results, DeepLabv3 + is preferable in cases where dentists don’t want to miss probable apical lesions since it has better recall than U-Net. On the other hand, the U-Net model is preferable in cases where dentists don’t want to take the risk of a false diagnosis because of its higher precision score.

Similarly, a model with a higher AUC is likely better at overall classification and distinguishing between lesion and non-lesion regions across varying thresholds but may not perform as well at a specific threshold in terms of the balance between precision and recall. A model with a higher F1-score is optimized for a specific threshold and strikes a better balance between detecting lesions and avoiding false positives at that threshold. If a dentist wants to focus on identifying all potential lesions (e.g., for initial screening), they should consider using the DeepLabV3 + model since it has a higher AUC in our experiments. However, if a dentist wants to accurately identify lesions with minimal false positives, they might prefer the U-Net model due to its higher F1-score.

Upon reviewing the literature, we found that some studies have used the U-Net model for apical lesion segmentation [2–3, 25]. We also found several studies that employed the DeepLabv3 + model to detect dental plaque and caries lesions [9, 22, 26, 27]. However, we did not come across any articles in which the DeepLabv3 + model was used for the segmentation of apical lesions.

In studies using U-Net, both cropped and uncropped panoramic images were used. Cropping images enhances the efficiency of the training and prediction process by reducing irrelevant or distracting information, allowing the model to focus on the most relevant areas. However, it is important to consider the potential drawbacks, such as information loss, bias, and reduced robustness. Striking the right balance between cropping and preserving relevant information is crucial for efficiently training machine learning models. Such efficient cropping requires expertise in this domain and can be time-consuming. If the images are cropped to obtain a region of interest (ROI) and train a more robust AI model, the dentists who wish to use such an AI tool would need to crop each image, a process that is prone to errors and can be time-consuming. In this study, we used uncropped images to avoid these disadvantages and to evaluate the performance of AI models on images without cropping.

Among the studies that used U-Net for the segmentation of apical lesions, Bayrakdar et al. cropped the panoramic images into four parts. Their recall, precision, and F1-score values were 0.92, 0.84, and 0.88, respectively, with an IoU value of 0.7 [2]. Krois et al. cropped the panoramic images based on the region of interest. They studied two different data sets. One dataset showed an F1-score of 0.54 and a precision of 0.64, while the other showed an F1-score of 0.32 and a precision of 0.63 [19]. Endres et al. cropped the panoramic images by 100 pixels on the upper and lower boundaries and 300 pixels on the left and right boundaries. They found a precision of 0.60 and an F1-score of 0.58 [25].

All three studies reported better precision and F1-score results than the U-Net and DeepLabv3 + results obtained in our study. However, since they did not report them, we were unable to compare our IoU, AUC, and recall metrics with Krois and Endres’ studies. On the other hand, Bayrakdar’s study obtained better results in all metrics than ours. One of the main reasons for such a difference in the results may be attributed to the use of cropping in these studies, which enhances the performance of the models, as previously mentioned. In addition to cropping, the number of images used for machine learning training plays a crucial role in the model performance. 470, 650, and 2900 images were used in the studies of Bayrakdar, Krois, and Endres respectively while in our study, only 260 images were used. Another important point that affects the model performance is the hyper-parameters used in the training. Unfortunately, there is no information about hyper-parameters used in Bayrakdar’s study except for the number of epochs which is 95. On the other hand, Krois used a linear combination of binary cross entropy and Dice loss functions to optimize the model with a 0.002 learning rate and 200 epochs while we only used binary cross entropy with a 0.001 learning rate and 100 epochs. Similarly, Endres used the Dice loss function in their training with 25 epochs. Although their learning rate is 0.001 as in our study, they reduced their learning rate with exponential decay. Most importantly, they used a different evaluation strategy by training 10 different models by dividing the training set into 10 subsets and using 9 of them for each model’s training. Then, they obtained the results by taking the mean output produced by the 10 constituent models which produced more robust results. Considering all these differences, it is understandable that the studies obtained different results than ours.

On the other hand, Song et al. used U-Net without any cropping, which is similar to our study. They reported precision, recall, F1-score, and IoU values of 0.744, 0.740, 0.742, and 0.5, respectively [3], while our U-Net scores were 0.55, 0.15, 0.23, and 0.56, and our DeepLabv3 + scores were 0.17, 0.24, 0.17, and 0.54, respectively. Although our precision, recall, and F1-score were lower than Song et al.’s results, we obtained better results on the IoU metric, which is the most commonly used evaluation metric in the image segmentation domain.

As stated above, we found no article in which the DeepLabv3 + model was used for the segmentation of apical lesions. But when we examined the articles comparing U-Net and DeepLabv3 + for caries lesion segmentation, we saw that Zhu et al. found a better F1-score in U-Net and better accuracy, precision, and recall metrics in DeepLabv3+ [22]. Zhang et al. reported a better recall in DeepLabv3 + and a better IoU with U-Net [27]. Chen et al. found better recall, precision, and IoU in U-Net than DeepLabv3+ [6]. The results of these studies indicate that, currently, U-Net and DeepLabv3 + cannot outperform each other across all metrics. Also, we obtained similar results in our study where DeepLabv3 + gave better recall and AUC results but U-Net obtained better IoU and precision results.

However, this study had several limitations, such as the number of patients, the use of a single panoramic radiography machine, and standardized parameters. Furthermore, the success of AI is closely linked to the accuracy of labeling (annotation), which depends on the skill of the practitioners. Although three experienced dental radiologists performed the labeling process, achieving entirely accurate labeling at a pixel level is not feasible. Future work may focus on evaluating the performance of DeepLabv3 + on larger, more diverse datasets, as well as using different radiography machines. Additionally, future studies may explore the use of other deep learning architectures for apical lesion segmentation and investigate the impact of cropping strategies on model performance. Moreover, due to the limited number of images, the validation set was not utilized in the training which might result in a low generalization and overfitting on the test set. We had planned to handle this difficulty by increasing images with data augmentation techniques. But, due to the limited computational power, we couldn’t achieve successful training. With higher computing devices, it will be possible to obtain more robust results with higher prediction performance both for U-Net and DeepLabV3+.

## Conclusion

In this paper, we comprehensively evaluated the DeepLabv3 + and U-Net models in the context of apical lesion segmentation. Our findings indicate that DeepLabv3 + outperforms the U-Net model by a substantial margin in terms of AUC and recall. However, it is behind the U-Net model in precision and F1-score, and there is no significant difference between the models in terms of the IoU metric. Overall, the DeepLabv3 + model outperforms U-Net in 2 of the 5 metrics, performs worse in 2 of the 5 metrics, and is equivalent in 1 of the 5 metrics. Although the DeepLabv3 + model does not outperform U-Net in all cases, it can still compete with the U-Net model in the segmentation of apical lesions based on these results. However, the current forms of the DeepLabv3 + and the U-Net model are not directly applicable in clinical diagnosis since their current results are not high enough due to limitations in the number of images available for training. On the other hand, with a larger dataset and a more detailed training process, these models could be used to improve clinical diagnosis and treatment planning.

## Data Availability

No datasets were generated or analysed during the current study.
